# Development of a GAL4-VP16/UAS trans-activation system for tissue specific expression in *Medicago truncatula*

**DOI:** 10.1371/journal.pone.0188923

**Published:** 2017-11-29

**Authors:** Amélie Sevin-Pujol, Mélanie Sicard, Charles Rosenberg, Marie-Christine Auriac, Agnès Lepage, Andreas Niebel, Clare Gough, Sandra Bensmihen

**Affiliations:** LIPM, Université de Toulouse, INRA, CNRS, Castanet-Tolosan, France; Università degli Studi di Milano, ITALY

## Abstract

Promoters with tissue-specific activity are very useful to address cell-autonomous and non cell autonomous functions of candidate genes. Although this strategy is widely used in *Arabidopsis thaliana*, its use to study tissue-specific regulation of root symbiotic interactions in legumes has only started recently. Moreover, using tissue specific promoter activity to drive a GAL4-VP16 chimeric transcription factor that can bind short upstream activation sequences (UAS) is an efficient way to target and enhance the expression of any gene of interest. Here, we developed a collection of promoters with different root cell layers specific activities in *Medicago truncatula* and tested their abilities to drive the expression of a chimeric GAL4-VP16 transcription factor in a trans-activation UAS: β-Glucuronidase (GUS) reporter gene system. By developing a binary vector devoted to modular Golden Gate cloning together with a collection of adapted tissue specific promoters and coding sequences we could test the activity of four of these promoters in trans-activation GAL4/UAS systems and compare them to “classical” promoter GUS fusions. Roots showing high levels of tissue specific expression of the GUS activity could be obtained with this trans-activation system. We therefore provide the legume community with new tools for efficient modular Golden Gate cloning, tissue specific expression and a trans-activation system. This study provides the ground work for future development of stable transgenic lines in *Medicago truncatula*.

## Introduction

Legume plants, or Fabaceae, are the third most important family of flowering plants in terms of number of species. This evolutionary success is believed to be largely due to the capacity of most of its 20,000 species to establish a symbiotic interaction, called nodulation, with soil bacteria called rhizobia [1]. Rhizobia can transform atmospheric di-nitrogen into a source of nitrogen that can be assimilated by the plant, inside a root organ called a nodule. Thanks to this symbiotic capacity, legume plants are rich in proteins and provide an important source of animal feed and human food. In this aspect, legumes are important players for food security and sustainable agriculture. Moreover, legumes are also able to form another root endosymbiotic interaction with arbuscular mycorrhizal (AM) fungi from the Glomeromycota phylum. This association notably provides plants with soluble forms of phosphate and sulfur. In contrast, the Brassicaceae *Arabidopsis thaliana* is not able to form an AM symbiotic interaction or form nodules with rhizobia. *Medicago truncatula* is a model legume plant used for many years to study root endosymbiotic interactions and several pathosystems have also been developed with root pathogens such as the oomycete *Aphanomyces euteiches* [2] and the soil borne bacterium *Ralstonia solanacearum* [3]. The constant efforts needed to better understand the root physiology and the genetic determinants of symbiotic and pathogenic interactions require new molecular tools adapted to legume roots.

Trans-activation tools such as the GAL4-VP16/ upstream activation sequences (UAS) system, originally developed in Drosophila [4], provide an opportunity to induce the expression of a transgene in a very specific tissue. Indeed, in this system, a chimeric transcription factor consisting of the yeast transcription factor GAL4 fused to the potent Herpes simplex virus activator VP16 can be driven by a specific promoter (in a so called “activator “line) and will bind specific GAL4 Upstream Activation Sequences (UAS) located upstream of the gene of interest in an “effector line” [5]. Transgenic plants carrying a UAS: reporter gene have also been largely used for enhancer trap strategies [6] [7] [8]. Due to technical limitations in high throughput whole plant transformation, such enhancer trap strategies are difficult to develop in *Medicago truncatula*. However, smaller scale plant transformation is possible [9] and the hairy root transformation system [10] provides a very efficient way to screen for transgene effects in legume roots. Another limitation to high throughput screening is the time needed for molecular cloning and obtaining a variety of modular constructs. Here, we describe new molecular tools adapted to the high throughput and modular “Golden Gate” cloning technique [11] to express any gene of interest under the control of a promoter with specific root cell layer activity. To test different root layer contexts, we adapted six tissue specific promoters originating from Arabidopsis, lupin or tomato and predicted to be specifically active in the root epidermis (*SlEXT1*), the root cortex (*AtCO2* and *AtPEP*), the root endodermis and the pericycle (*AtCASP1* and *LaSCR1*) or phloem companion cells (*AtSUC2*). We also designed a GAL4-VP16/ UAS cloning module to compare “direct” promoter: reporter fusions of these specific promoters to a trans-activation version. Although it was slightly more difficult to obtain hairy roots showing high levels of GUS reporter gene activity using the trans-activation system, this study lays the ground work for developing a GAL4-VP16/ UAS trans-activation system in *M*. *truncatula* roots and provide new tools that should be useful for the legume community.

## Material and methods

### Plant growth and transformation

Surface sterilized *Medicago truncatula* cv. Jemalong A17 seeds were sown on agar plates and placed for 3 days in the dark at 4°C, then left overnight at 25°C to germinate.

For root transformation, we used ARqua1 *Agrobacterium rhizogenes* as described by Boisson-Dernier [10]. Following hairy root transformation, seedlings were grown vertically on Fahraeus medium supplemented with 25 μg/mL kanamycin in a growth chamber at 21°C (16 h light/8 h dark cycles) for one week and then for 2 weeks at 25°C (16 h light/8 h dark cycles). *A*. *rhizogenes* transformed roots were selected by *DsRED* expression carried by the modified pCAMBIA2200 binary vector, pCAMBIA-CR1, and by kanamycin resistance.

### DNA constructs

For the Golden Gate cloning strategy [11]: promoters were amplified as “AB” blocks, β-glucuronidase (GUS) was amplified either as a “BD” block for direct promoter: GUS fusion or as a “CD” block for UAS:GUS fusions, GAL4-VP16 and UAS were obtained as “BN” and “NC” blocks using the primers listed in S1 Table. The “N” chosen adapter sequence corresponds to TTCA. Matrices for *pSlEXT1* and *pAtCO2* amplification were plasmids described in [12]. Amplification of the *LaSCR1* promoter region was obtained as described in [13]. The promoter regions (see Table 1) of *AtPEP* and *AtSUC2* were amplified from *Arabidopsis thaliana* Col0 genomic DNA and the promoter of *AtCASP1* from the gateway cloning vector pEN_R4_CASP1pro-L1 kindly provided by B. Péret (BPMP, Montpellier, France). An “NC” block containing the NOS terminator and the 5XUAS fragment together with a minimal promoter was synthetized by Eurofins Genomics following the sequence described in pEN_L4_UAS_R1 (https://gateway.psb.ugent.be/vector/show/pEN-L4-UAS-R1/search/index/). A synonymous point mutation in the GAL4 coding sequence was introduced to remove the internal BsaI restriction site. The pCAMBIA_CR1 vector is fully described in the results.

**Table 1:** References and expected tissular expression of all tested promoters.

Gene promoter name	Reference	Origin of the promoter DNA fragment	Size/ presence of BsaI site	Expected tissular expression in roots
***SlEXT1***	[17] [18]	Tomato (*Solanum lycopersicum*)	1.1 kb / No BsaI site	Epidermis, young root hairs
***AtCO2***	[19]	Arabidopsis (*Arabidopsis thaliana*)	560 bp / no BsaI site	Cortex (basal meristematic zone and above)
***AtPEP***	[20]	Arabidopsis (*Arabidopsis thaliana*)	1.7 kb / one BsaI site	Cortex, whole root
***AtCASP1***	[21] [22]	Arabidopsis (*Arabidopsis thaliana*)	1.2 kb / no BsaI site	Endodermis
***LaSCR1***	[23]	White lupin (*Lupinus albus*)	1.2 kb / one BsaI site	Endodermis and pericycle
***AtSUC2***	[24]	Arabidopsis (*Arabidopsis thaliana*)	2.1 kb / no BsaI site	Phloem companion cells

### Histochemical analysis

Histochemical staining for β-glucuronidase (GUS) activity was performed according to [14]. Transgenic roots were first fixed for 30 min in 0.5% paraformaldehyde solution buffered at pH 7 and rinsed prior to incubating in the dark for 3 to 6 hours at 37°C in a pH 7 phosphate buffer containing 2.5% 5-bromo-4-chloro-3-indolyl-beta-D-glucuronic acid (X-gluc, Biosynth.) as substrate. Tissues were subsequently rinsed in phosphate buffer and fixed in a 1.25% glutaraldehyde solution buffered at pH 7 for 2 h at room temperature.

### Microscopy studies

For root sections, stained transgenic roots were embedded in Technovit 7100 resin (Heraeus Kulzer, Wehrheim, Germany) and 10 μm sections were obtained as described in [13]. The sections were counter-stained with 0.05% ruthenium red for 10 minutes and observed with an Axioplan 2 microscope (Zeiss).

## Results

### New tools for modular cloning strategies in *Medicago truncatula*

**Fig 1:**
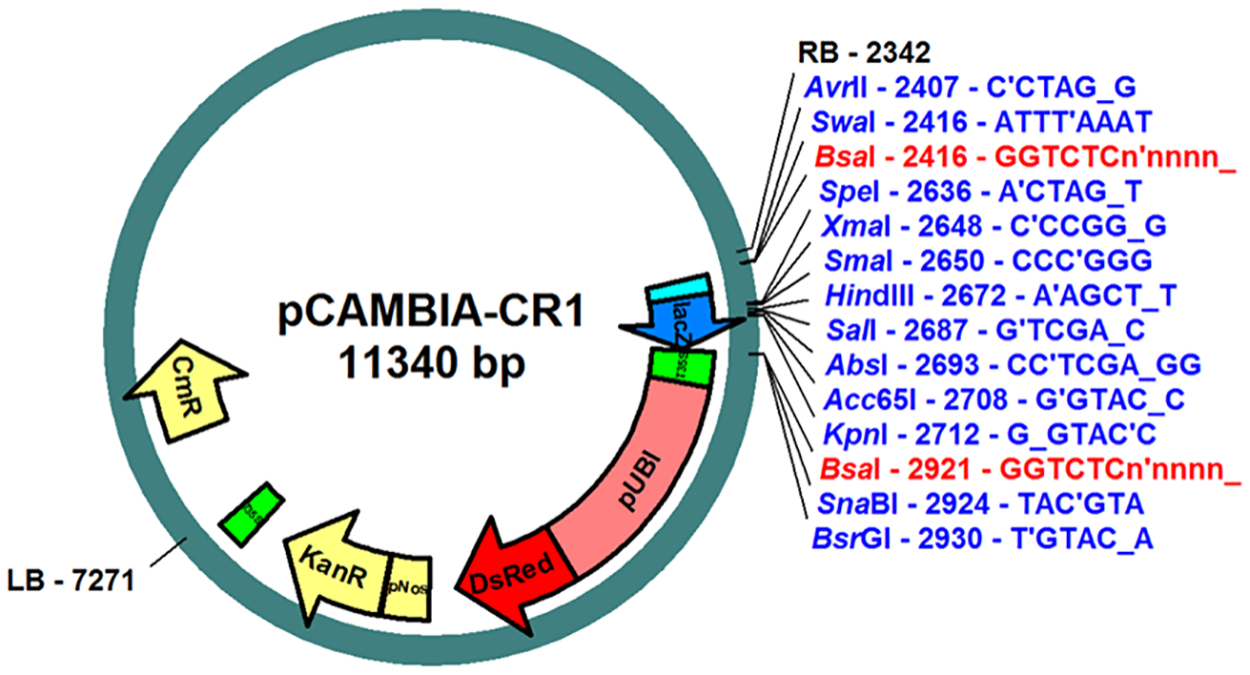
New pCambia Golden Gate vector. Schematic backbone of the pCAMBIA_CR1 vector showing the BsaI cloning sites (in red) that allow insertion of the oriented blocks using the Golden Gate strategy. Cloning sites (single cutter restriction enzymes or BsaI cloning sites) disrupt the LacZ gene (blue arrow) upon cloning, allowing blue/ white screening with the X-Gal substrate. For *E*.*coli* selection, a chloramphenicol (Cm) resistance gene can be used (yellow arrow outside the T-DNA fragment). A kanamycin resistance (kanR) gene, driven by a NOS promoter (yellow box and arrow), enables both selection for the presence of the plasmid in *A*. *rhizogenes* and transformed roots on selective medium. The T-DNA contains a *pAtUbi*:*DsRED* selection gene (red box and arrow) that allows detection of transformed roots using DsRED fluorescence. RB/LB: T-DNA right border and left border.

To rapidly test numerous constructs, we first developed a modular system to clone rapidly and in an oriented manner multiple blocks of promoter, reporter or trans-activation modules and test these constructs in the *M*. *truncatula* hairy root system. To do so, we took advantage of the Golden Gate cloning system [11]. As this system relies on the type II-s restriction enzyme BsaI restriction sites, we first removed such sites from the pCAMBIA2200 binary vector. To eliminate the unique *Bsa*I site present in the pCAMBIA2200 backbone, a BsaI/NheI fragment containing the replication origin *DnaA* was removed and replaced by the corresponding PCR fragment in which one base of the native BsaI recognition site was modified. Most of the T-DNA region was then removed by a PvuI digest followed by autoligation. A new T-DNA region, consisting of the right border (RB) sequence, the *LacZ* gene from pBlueScript KS(+) flanked by two BsaI sites, a fragment originating from the pK7WIWG2-UBQ120-Red containing the 35S terminator and the DsRed marker gene under the control of a *pAtUbiquitin* promoter [15], the *NptII* gene under the control of the pNos promoter and the left border (LB) sequence was thus created (Fig 1). This new binary vector, which we named pCAMBIA_CR1, allows efficient transformation of *Medicago* either via *Agrobacterium rhizogenes* or *A*. *tumefaciens*, just as the original pCAMBIA2200. Transformed cells can be selected by addition of kanamycin, and/or visualized by DsRed fluorescence.

The presence of the two BsaI sites allows multiple cloning in a single step by the Golden Gate cloning method, but the vector is also suitable for classical cloning, due to several unique restriction sites in the LacZ fragment (Fig 1). The X-Gal staining can be used to identify recombinant colonies, for either Golden Gate or classical cloning. Furthermore, due to the presence of unique restriction sites on both sides of the BsaI sites, classical and Golden Gate cloning can be carried out in combination (as described in [16]).

**Fig 2:**
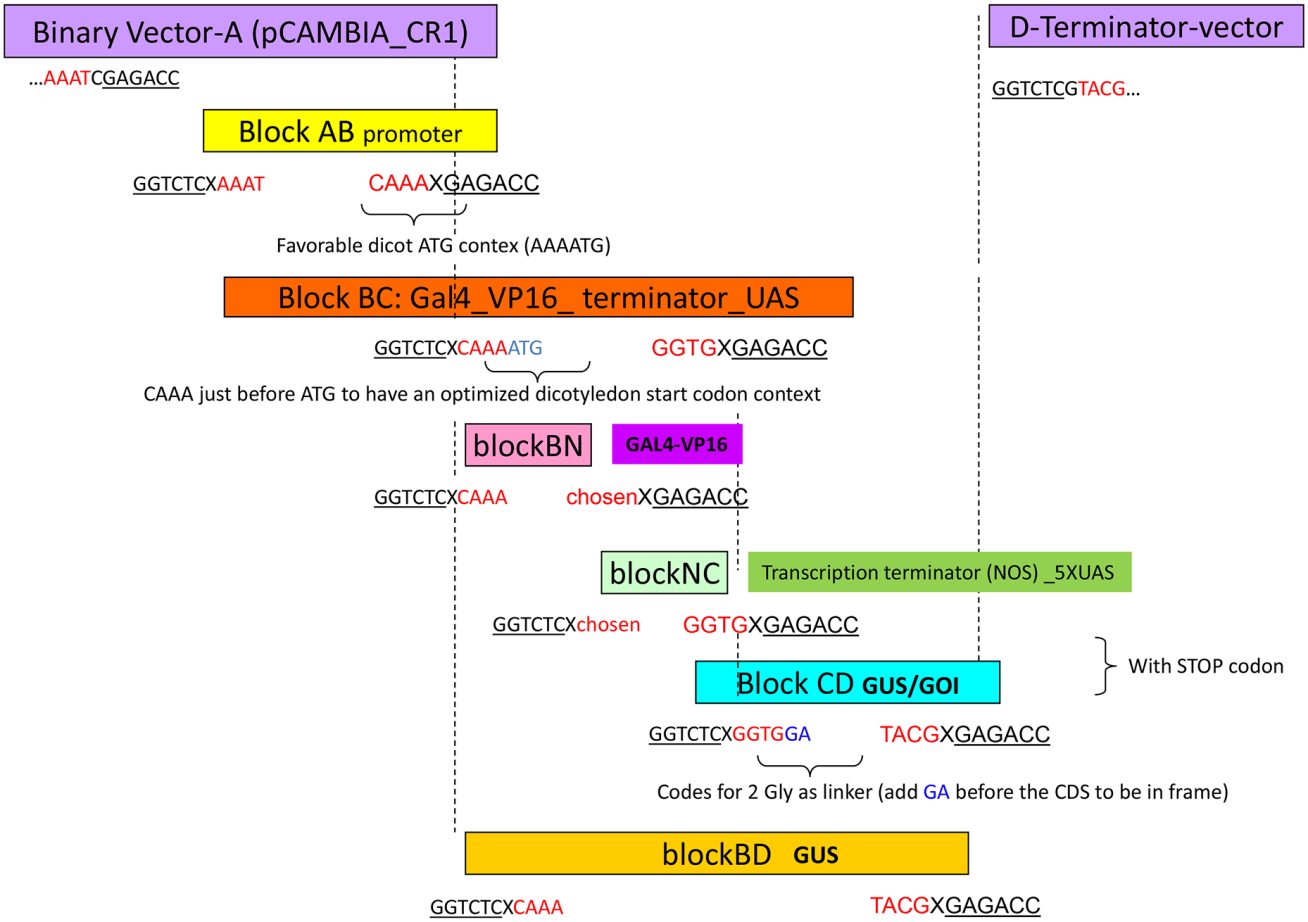
GoldenGate cloning strategy and consensus “sticky end” adapters used. Schematic representation of the consensus sequences used as “sticky ends” for oriented cloning of each specific block. The pCAMBIA_CR1 binary vector backbone is shown in purple. Here, “AB” blocks are promoter regions, the “BC” block was separated as “BN” and “NC” fragments (where N is a chosen sequence, here TTCA) for the GAL4-VP16 chimeric transcription factor and for the “transcriptional terminator 5xUAS_minimal promoter” blocks, respectively. The β-glucuronidase (GUS) coding region was introduced as a CD block for UAS constructs or BD block for the direct promoter fusions. Note that the “B” adapter was designed to provide an optimized dicotyledon start codon context and the “C” adapter to provide a linker for in frame tag fusions, respectively. X is a chosen spacer nucleotide that will be removed after BsaI digeston. GOI: gene of interest.

For subsequent Golden Gate cloning in pCAMBIA_CR1, promoter regions, coding sequences (CDS) and tags were amplified with primers flanked by BsaI sites designed to generate standardized specific 5' protruding ends and cloned as individual modules in pGemT, pJeT or pBlueScript cloning vectors (all carrying an ampicillin resistance gene). Protruding ends are designated as A (corresponding to an AAAT sticky end), B (corresponding to CAAA), C (corresponding to GGTG) and D (corresponding to TACG) (Fig 2). Each module is flanked by A-B, B-C, C-D or B-D sticky ends, allowing their cloning in a defined order into the pCAMBIA-CR1 binary vector, for which BsaI digestion generates A and D sticky ends (see Fig 2). We next used this Golden Gate methodology to rapidly clone various promoters in different reporter systems.

### Tissue specific promoters and development of a trans-activation tissue specific system

To drive tissue specific expression in *M*. *truncatula* roots, we chose six different promoters that were amplified from the genomes of the species of origin (Table 1): the promoter from the extensin gene from *Solanum lycopersicum* (*SlEXT1*) was used for root epidermal expression, the *Arabidopsis thaliana AtCO2* and *AtPEP* were chosen for cortical expression and the *AtCASP1* was used for endodermis/ pericycle expression. For these four promoters, both “classical” promoter:GUS fusions and trans-activation constructs (see below) were made to compare their tissue specific activity *in planta*. In addition, promoters from the *Lupinus albus SCARECROW* 1 (*LaSCR1)* and the sucrose transporter *AtSUC2*, expected to be active in the endodermis/pericycle and in phloem companion cells, respectively, were used for promoter:GUS fusions but were not tested for the trans-activation system (S1 Fig).

To test their tissue specific activity, promoters were introduced as A-B modules and a β-glucuronidase enzyme (GUS) coding sequence, containing an intron to prevent bacterial expression [25], was used as a reporter gene and cloned as a “BD” or “CD” block. In parallel, we used a two-step cloning strategy to generate a “BC” block with a DNA-binding domain of the yeast GAL4 transcriptional activator fused to the potent Herpes simplex VP16 transcriptional activation domain [26], together with a NOS terminator sequence and five Upstream Activation Sequences (UAS) specific for GAL4 (Fig 2, see Material and methods). These UAS are similar to those used for trans-activation studies in *Arabidopsis thaliana* [27]. In this manner, we obtained two types of constructs: a binary vector with a promoter:GUS construct (by combining AB and BD blocks) on the one hand, and a promoter: GAL4-VP16/ 5xUAS:GUS, on the other hand. These two types of constructs were introduced in *M*. *truncatula* roots using *Agrobacterium rhizogenes* transformation and their root tissular expression patterns were compared among many (33–126) independent transformed roots.

### Comparison of root tissular activities of promoter:GUS and trans-activatable constructs

Transformed roots were selected on the basis of the fluorescence conferred by the DsRed marker gene (as shown on Fig 1). Table 2 summarizes the number of independent roots and their GUS expression patterns observed for the promoter:GUS and GAL4-VP16/ UAS transactivation constructs, respectively. We distinguished three types of expression patterns: i) “GUS-”roots were fluorescent for the DsRed reporter, so likely transformed, but did not display any visible GUS activity; for the GUS positive roots, we distinguished ii) those only showing the expected (specific) macroscopic tissular expression pattern and iii) those that showed non specific staining (for instance, cortical expression for the *SlEXT* epidermal promoter or expression in root hairs for the *AtCO2* and *AtPEP* cortical promoters).

**Table 2:** Comparison of GUS staining efficiency and tissue specificity of promoter:GUS and transactivatable (GAL4-VP16/ UAS) GUS constructs.

Construct	Total number of plants tested	Specific GUS + number of plants (and %)	Non specific GUS + number of plants (and %)	GUS- number of plants (and %)
**pSlEXT:GUS**	83	64 (77%)	16 (19.3%)	3 (3.7%)
**pSlEXT:GAL4-VP16/ UAS_GUS**	68	17 (25%)	27 (39.7%)	24 (35.3%)
**pAtCO2:GUS**	49	39 (79.6%)	0	10 (20.4%)
**pAtCO2: GAL4-VP16/ UAS_GUS**	33	7 (21.2%)	3 (9.1%)	23 (69.7%)
**pAtPEP:GUS**	122	87 (71.3%)	31 (25.4%)	4 (3.3%)
**pAtPEP: GAL4-VP16/ UAS_GUS**	39	15 (38.5%)	16 (41%)	8 (20.5%)
**pAtCASP:GUS**	126	93 (73.8%)	29 (23%)	4 (3.2%)
**pAtCASP: GAL4-VP16/ UAS_GUS**	39	21 (53.8%)	7 (18%)	11 (28.2%)

**Fig 3:**
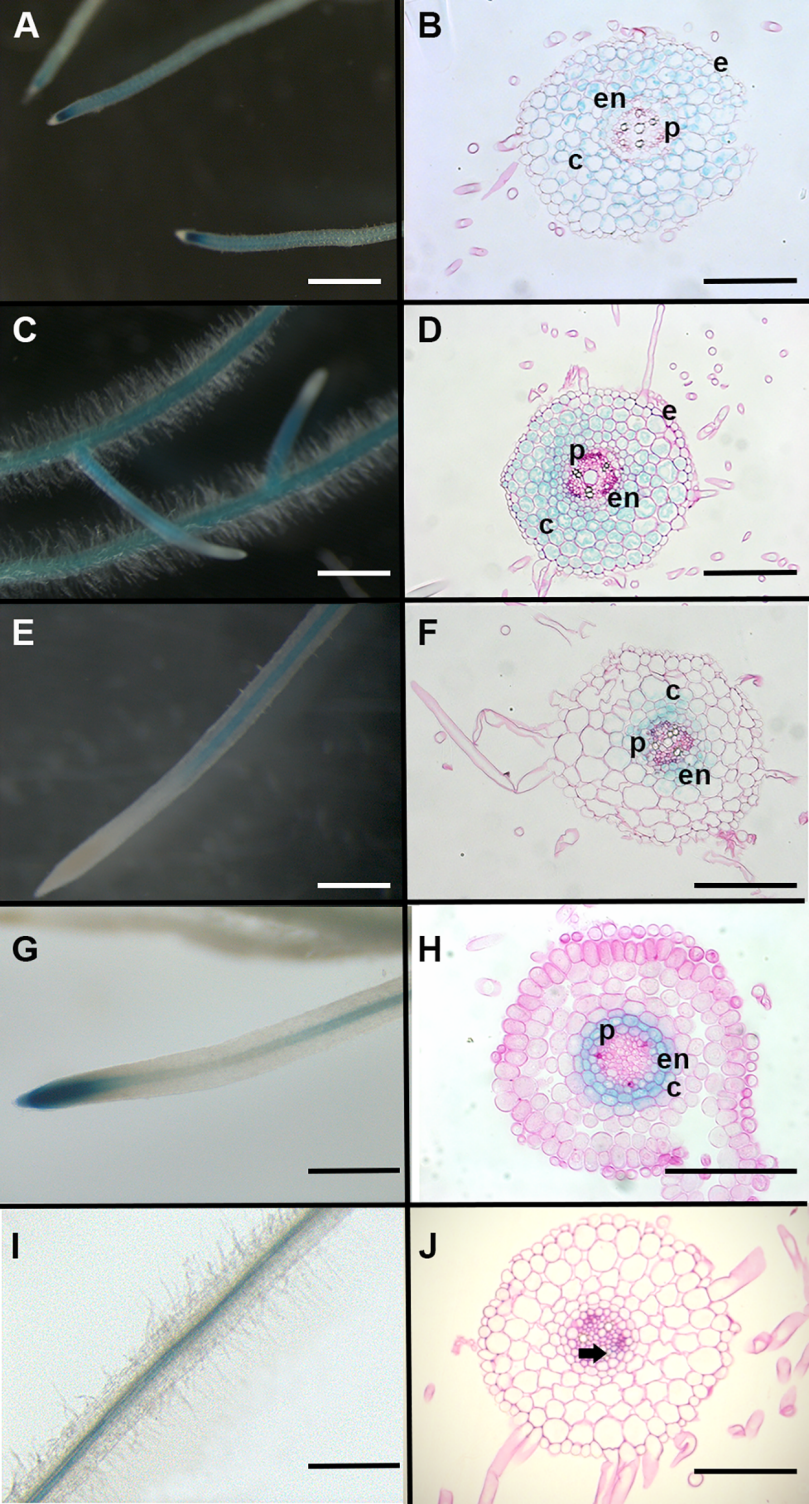
Whole root and root section patterns of tissular GUS activity obtained in promoter:GUS fusions. Whole *M*. *truncatula* transgenic roots and representative root sections showing GUS activity driven by the (A,B) *pAtCO2*, (C,D) *pAtPEP*, (E,F) *pAtCASP1*, (G,H) *pLaSCR1* and (I,J) *pAtSUC2* promoters using direct fusions with GUS coding sequence. GUS staining is shown in blue. Scale bar is 1 mm in (A,C,E,G,I) and 100 μm in (B,D,F,H,J). e: epidermis; c: cortex; en: endodermis; p: pericycle. Arrow head in (J) marks phloem companion cell.

First, observation of whole roots transformed with promoter:GUS fusions cloned in our Golden Gate system showed that the cortical promoters *pAtCO2* (Fig 3A) and *pAtPEP* (Fig 3C) and the endodermal promoter *pAtCASP1* (Fig 3E) displayed the expected root tissue specific activity in 70–80% of transformed *M*. *truncatula* roots (Table 2). We also observed that *pLaSCR1* was active in the endodermis but also in pericycle cells (Fig 3G and 3H; 38/40 tested plants) and the *pAtSUC2*:GUS fusion displayed the expected phloem companion cells expression (Fig 3I and 3J; 17/40 tested plants for whole roots). The *AtPEP* promoter activity appeared to have a complementary expression pattern to the *AtCO2* promoter in whole roots. Indeed, while *AtCO2* promoter activity was stronger in basal meristems (and in dividing cortical cells, as described in [12]) and weaker in older parts of the root (Fig 3A), the *AtPEP* promoter activity appeared stronger in older parts of roots, but was absent in the basal meristem area (Fig 3C).

**Fig 4:**
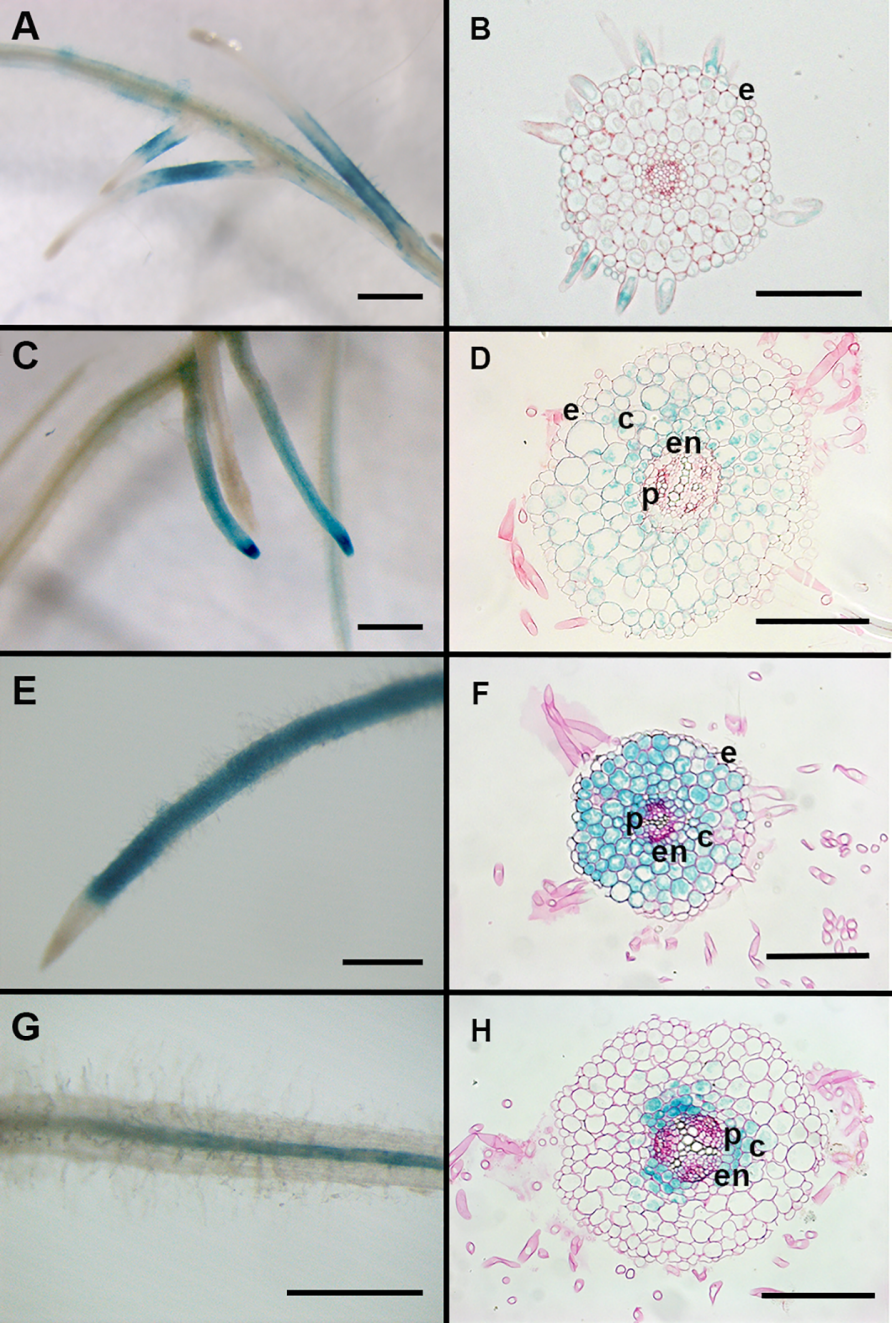
Whole roots and root sections showing patterns of tissular GUS activity obtained in the GAL4-VP16/UAS trans-activation system. Whole *M*. *truncatula* transgenic roots and representative root sections (10 μm) showing GUS activity driven by the (A,B) *pSlEXT1*, (C,D) *pAtCO2* (E,F) *pAtPEP* and (G,H) *pAtCASP1* promoters using GAL4-VP16/UAS transactivatable system. GUS staining is shown in blue. Scale bar is 1 mm in (A, C, E,G) and 100 μm in (B,D,F,H). e: epidermis; c: cortex; en: endodermis; p: pericycle.

Then, to address the efficiency and specificity of the GAL4-VP16/ UAS trans-activation system, we compared the patterns observed in promoter:GUS fusions to the same promoter driving GAL4-VP16 expression upstream of an UAS:GUS sequence. To do so, we used a single transgene that contained the promoter:GAL4-VP16 and UAS:GUS sequences, as described above. The DsRed marker gene present in the pCAMBIA-CR1 binary vector enabled selection of transformed roots that were harvested and stained for GUS activity. We could obtain a significant number of roots with specific GUS staining (see Table 2) using the *pSlEXT1* (Fig 4A), *pAtCO2* (Fig 4C), *pAtPEP* (Fig 4E) or *pAtCASP1* (Fig 4G) promoters driving GAL4-VP16 in our trans-activation system. While promoter:GUS fusions gave very few transformed roots without any GUS staining (around 3%, except for *pAtCO2* where we reached approximately 20%), this proportion was higher in promoter:GAL4-VP16/UAS:GUS constructs (around 20–30% except for *pAtCO2* driven GAL4-VP16, where we reached 69%). In roots where we could detect GUS staining, we observed that the proportion of expected tissue specific expression pattern by a given GAL4-VP16/UAS:GUS driven construct was much lower than those obtained with the equivalent promoter:GUS fusion. The *pAtCO2* and *pAtCASP1* driven GAL4-VP16/UAS:GUS expression appeared the most efficient in terms of specificity, compared to *pSlEXT* and *pAtPEP* (Table 2).

For some of the transformed roots (both for classical constructs and trans-activation systems) that displayed whole root GUS pattern with the expected tissue specificity, we made transverse sections to further confirm their specific tissular expression. As shown in Figs 3 and 4, root sections revealed that *AtCASP1* promoter activity was not limited to the endodermis but was also found in the pericycle and inner cortex (Figs 3F and 4H). Interestingly *pAtCASP1* activity often appeared stronger in cells opposite xylem poles (Figs 3F and 4H). In contrast, the expression pattern of the *LaSCR1*:*GUS* construct was more homogenous and appeared more restrained to the endodermis, even if it was also detected in the pericycle (Fig 3H). Moreover, root sections showed that *pAtPEP* could also display some activity in the root endodermis in *M*. *truncatula* roots (Figs 3D and 4F) while pAtCO2 did not (Figs 3B and 4D). In these root sections, we observed similar tissular expression in roots transformed with a trans-activation construct (with promoters driving GAL4-VP16 in front of a UAS:GUS reporter gene) compared to those observed with classical promoter:GUS fusions (Figs 3 and 4). Although we did not quantify their expression levels, we noticed that GUS staining obtained with the trans-activation system appeared stronger than that observed with the promoter:GUS constructs (Compare Figs 3D and 4F, Figs 3F and 4H).

## Discussion

### New tools for modular cloning

In this work, we have successfully adapted a pCAMBIA binary vector and tissue specific promoter sequences for the Golden Gate cloning system and created a module for GAL4-VP16/ 5xUAS trans-activation assays. We created a new set of vectors, promoters and reporter genes that can be easily assembled. A pCAMBIA_CR2 vector, with deletion of the DsRed reporter is also available for cloning of fluorescent tags or other reporter genes. This provides new tools for highly efficient cloning and tissue specific expression in *M*. *truncatula* roots that are now available to the legume community. Additional promoters of interest can now be easily adapted to this cloning system by amplifying with the dedicated “A” and “B” sticky ends we have described. In doing so, one should however check that no more than one BsaI site is present in the desired promoter sequence (for only one remaining BsaI site, the problem is easily overcome by adding a final ligation step in the Golden Gate cloning procedure). All BsaI sites were removed from the coding sequences used for our constructs, such as the one in the GAL4 gene, by PCR amplification with primers containing the desired substitution.

Through our extensive characterization of transformed roots (between 33 and 126 independent roots analyzed for each construct) and thin root sections for several of them, our study provides detailed tissular activity analysis for six promoters in *Medicago truncatula* roots, including the previously un-described promoter *AtPEP*. The other promoters used in this study have previously been tested in *M*. *truncatula* [12] [28] [13] [29], and some (such as *pSlEXT1* and *pAtCO2*) have also been tested in *Lotus japonicus* roots [30]), confirming that transferring promoter activity from Arabidopsis to legume roots works well. However, our refined level of analysis gave more precise information on several promoter activities. For example, in our hands, the *LaSCR1* promoter appeared more specific for expression in the endodermis than the *AtCASP1* promoter since, in contrast to what was described by Xiao et al. [28], we consistently observed *pAtCASP1* activity in the innermost cortical cell layers of *M*. *truncatula* (Fig 4A and 4B). Moreover, *pAtCASP1* appeared more active in cells opposite xylem poles. However, none of the promoters we tested displayed a tissular activity fully restricted to the endodermis. The *AtPEP* promoter was consistently active in all cortical cell layers, and often in the endodermis (Fig 4C and 4D). The high level of activity of the *AtPEP* promoter along the primary root makes this promoter a better option than the previously used *pAtCO2* for a strong expression in *M*. *truncatula* cortical layers with exclusion from the epidermis in tissue specific complementation assays [12, 31]. Interestingly, most of the promoters we used here were also shown to be active upon rhizobium inoculation and especially in nodule tissues ([12] [28] [29] [30], S1 Fig), indicating that our tools can also be useful for nodulation studies.

### Interest of tissue specific and trans-activation expression systems

Promoters with tissue-specific activity are very useful to address cell-autonomous and non cell autonomous functions of candidate genes. Although this strategy is widely used in *Arabidopsis thaliana*, its use to study tissue-specific regulation of symbiotic interactions has only recently been exploited ([12] [31]). However, transgenic roots obtained with such tissue-specific complementations can display variable levels of production of the gene of interest (GOI), due notably to genome insertion effects. Therefore, there is a need for “stable” transgenic plants that can guarantee an homogenous level of tissular expression of your favorite gene. In this respect, trans-activation constructs can be used, by crossing two different well characterized transgenic lines: one providing the tissue specific expression of the GAL4-VP16 transcription factor (“activator” line) and the second one the UAS:GOI target (the “effector” line). Moreover, the expression of the highly active GAL4-VP16 transcription factor together with the use of multiple upstream activation sequences (UAS) can enhance the level of activation of the GOI compared to the same tissue specific promoter alone [5]. This strategy has proven effective in many Arabidopsis studies to assess developmental functions of candidate genes (see [32]). Until now, no such trans-activation system had been tested in legume roots such as *Medicago truncatula*. In this study, we have shown that the GAL4-VP16/ 5xUAS trans-activation system can work in *M*. *truncatula* roots, despite some variability depending on the promoter used to drive the GAL4-VP16 transcription factor.

### Comparison of direct promoter:Reporter fusions and trans-activation expression systems

When we compared the efficiency and tissue specificity of the promoter:GUS and promoter:GAL4-VP16/UAS:GUS constructs in the hairy root transformation system, we observed that the trans-activation system was less active than the promoter:GUS fusions, with many more transformed roots that did not display any GUS expression. Moreover, non specific GUS activity was more often detected in the trans-activation system, suggesting that these types of trans-activation constructs could be more prone to genome insertion effects, for instance. This “genome effect” could be related to methylation of the UAS transgene in *M*. *truncatula*. Indeed, it was previously shown in tobacco that GAL4 DNA binding capacity can be impaired by plant chromatin methylation [33]. This could explain the high level of transformed roots showing no GUS staining. Some promoters appeared less problematic than others for the specificity aspect. For instance, *AtCASP1* and *AtCO2* promoters gave high proportions of specific GUS expression patterns in the GAL4-VP16 system, although roots transformed with the *pAtCO2* construct gave a high number of roots with no GUS activity (Table 2). In contrast, for the *pAtPEP* driven trans-activation system, the chance of getting a specific tissular expression was similar to that for a non specific pattern but with a high frequency (around 40% of transformed roots in both cases). This suggests that, despite the different root organization of Arabidopsis, which displays only one cortical cell layer versus three to four in *M*. *truncatula*, Arabidopsis promoters are suitable for tissue-specific use in *M*. *truncatula*. In this respect, this trans-activation tool remains suitable for “stable” transgenic plants that would be selected for high and specific reporter gene activity.

### How could efficiency and tissue specificity be improved?

In our conditions, the GAL4-VP16 /5x UAS trans-activation system was thus working but with a rather low efficiency. Despite this low efficiency, the frequency of the transformed roots displaying a proper tissular pattern and high level of GUS expression is still high enough (from 21% for the *pAtCO2* driven construct up to 54% for the *pAtCASP1* driven construct) to enable selection of useful stable transformant lines. In our hands, efficiency of Medicago whole plant transformation is around 10% when using kanamycin selection and pCAMBIA vectors. Therefore, we can reasonably expect to obtain between 2 to 5 useful transformant lines for each construct. Generating stable transformant lines is a long time process (approximately one year to regenerate T0 and obtain seeds) [9]. Therefore, although our constructs could all be used as they are, one could also think of different ways to improve the system.

On top of genomic context and transgene insertion effects, other reasons could impact transgene expression and different improvements of the technique can be suggested. One limitation could be the quantity of the chimeric GAL4-VP16 transcription factor produced. Maybe the amount of GAL4-VP16 required to trans-activate the UAS:GUS construct was not optimal in many cases. This could explain why many of the roots transformed with the trans-activation construct did not show any GUS activity. To overcome this problem, stable transgenic lines displaying high levels of GAL4-VP16 protein accumulation with the different promoters tested could be selected. A recent study in *Caenorhabditis elegans* showed that all components of the GAL4/UAS trans-activation system can be optimized to enhance its efficiency [34]. Indeed, Wang and collaborators showed that using a form of VP16 with four tandem repeats (named VP64), increasing the number of UAS repeats (from 5 to 15) and using another GAL4 protein from a yeast strain more adapted to grow around 23–25°C could significantly enhance the efficiency of the GAL4-VP16/UAS trans-activation system in *C*. *elegans*. However, they only tested the combination of separate UAS:GFP and promoter:GAL4-VP16 constructs. Another reason to explain our results could be that having the UAS sequences so close to the GAL4-VP16 gene expression may not be optimal. Maybe separate constructs with the promoter:GAL4-VP16, on the one hand, and the UAS:GUS (or gene of interest, GOI) on the other hand would be best. We tested a vector carrying both components to be sure that all the necessary elements were present and to avoid double transformation issues, but having separate constructs might be better. In this respect, such a trans-activation system could be useful for the *M*. *truncatula* community if we could obtain “elite” lines with good GAL4-VP16 production and tissue specific activity (to be tested with an internal UAS:GFP marker, for instance). These lines could then be used for retransformation/ crossing with other UAS:GOI constructs.

## Supporting information

S1 Fig Thin section profile of GUS activity in *LaSCR1*:*GUS Medicago truncatula* nodules. Root cross sections showing GUS activity driven by the *LaSCR1* promoter fusions in nodules of *M*. *truncatula*. GUS staining is shown in blue. 10 μm cross-sections were counter stained with ruthenium red. p: pericycle, en: endodermis. Bar = 100 μm.
(PDF)

S1 Table Primers used for Golden Gate cloning. (XLSX)
