# Supplementary material for: Development of a GAL4-VP16/UAS trans-activation system for tissue specific expression in Medicago truncatula
Source: PLoS One. 2017 Nov 29;12(11):e0188923. doi: 10.1371/journal.pone.0188923 (PMC5706680; doi:10.1371/journal.pone.0188923)
Supplement: S1 Fig — Root cross sections showing GUS activity driven by the LaSCR1 promoter fusions in nodules of M. truncatula. GUS staining is shown in blue. 10 μm cross-sections were counter stained with ruthenium red. p: pericycle, en: endodermis. Bar = 100 μm. (PDF) [file pone.0188923.s001.pdf]

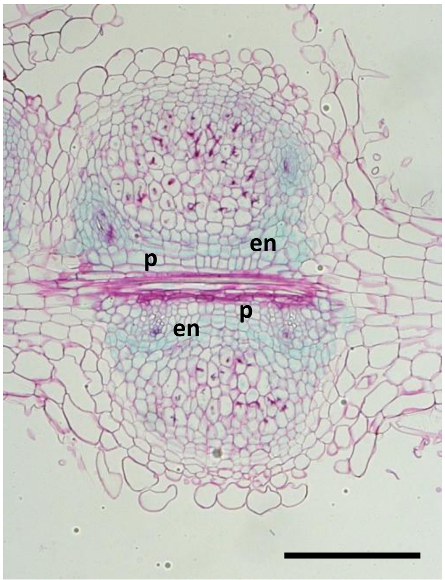

S1 Figure. Thin section profile of GUS activity in *LaSCR1:GUS Medicago truncatula* nodules

Root cross sections showing GUS activity driven by the *LaSCR1* promoter fusions in nodules of *M. truncatula.* GUS staining is shown in blue. 10 µm cross-sections were counter stained with ruthenium red. p: pericycle; en: endodermis. Bar= 100 µm
